# The Sixteenth International Medical Congress

**Published:** 1909-02-13

**Authors:** 


### THE SIXTEENTH INTERNATIONAL MEDICAL CONGRESS.

The sixteenth International Medical Congress, under the patronage of H.M. the Emperor Francis Joseph, will be held at Budapest from August 29 to September 4, 1909. The office is at Budapest (Hungary), VIII. Esterházy-utcza 7, and the Secretary-General is Dr. E. Grosz. We are requested to call the attention of speakers at the Congress to the announcement that the manuscripts of their addresses should be despatched by February 28, 1909, at the latest, to the office of the Congress. We are also asked to remind them that the subscription to this scientific gathering is twenty-five crowns in Austro-Hungarian currency, which sum may be sent by Post Office money order to Professor Dr. de Elischer, Treasurer of the Congress, VIII. Esterházy-utca 7, Budapest.

